# Kentucky Surgery

**Published:** 1853-04

**Authors:** 


### SELECTIONS

FROM

### FOREIGN AND HOME MEDICAL JOURNALS.

---

*Kentucky Surgery*. From the Report of Prof. Gross to the Kentucky Medical Society.—*Ovariotomy*. — To Kentucky belongs the honor of having furnished to the world the first case of extirpation of the ovary, for organic disease of this organ. This honor is justly and exclusively due to the late Dr. Ephraim McDowell, of Danville. From a paper published by this gentleman in the seventh volume of the Philadelphia Eclectic Repectory, it appears that his first operation was performed in December, 1809. It is not known, with any degree of certainty, how often Dr. McDowell repeated this operation; his published cases amount only to five, but there is reason to believe, from what I have learned from his nephew, Dr. Wm. A. McDowell, that he performed it not less than thirteen times.

During the progress of my labor, as Chairman of the Committee on Surgery, of this Society, I have, in consequence of letters addressed to various gentlemen in Kentucky, Ohio, and Tennessee, been made acquainted with the particulars of three cases more, which, added to those published by Dr. Ephraim McDowell himself, increase the aggregate to eight. It is to be deeply regretted that

Dr. McDowell did not keep a record of his operations, or communicate the results to his professional brethren. Such a contribution would not only have greatly enhanced his reputation as a bold and original surgeon, but it would have conferred an inestimable boon upon suffering humanity.

As the operations in question reflect the highest credit, not only upon Kentucky, and upon Kentucky's illustrious surgeon, but upon the United States, and as they have been mainly, if not exclusively, instrumental in directing the attention of the profession, both in America and in Europe, to a subject which has, of late years, elicited so much interest, research and skill, it is proper that I should give a brief analysis of them, in order, more especially, that the world at large may know what knowledge and science, when aided by intrepidity and dexterity, may accomplish even in a backwoods settlement of Kentucky. Dr. McDowell's first three cases are published in the seventh, and the last two in the ninth, volume of the Philadelphia Eclectic Repertory.

Dr. McDowell's *first operation* was performed upon Mrs. Crawford, of Ken ucky, in December, 1809. The tumor inclined more to one side than the other, and was so large as to induce her professional attendant to believe that she was in the last stage of pregnancy. She was affected with pains similar to those of labor, from which she could find no relief. The wound was made on the left side of the median line, some distance from the outer edge of the straight muscle, and was nine inches in length. As soon as the incision was completed, the intestines rushed out upon the table; and so completely was the abdomen filled by the tumor that they could not be replaced during the operation, which was finished in 25 minutes. In consequence of its great bulk, Dr. McDowell was obliged to puncture it before it could be removed; he then threw a ligature around the fallopian tube, near the uterus, and cut through the attachments of the morbid growth. The sac weighed seven pounds and a half, and contained fifteen pounds of a turbid, gelatinous looking substance. The edges of the wound being brought together by the interrupted suture and adhesive strips, the woman was placed in bed and put upon the antiphlogistic regimen. "In five days," says Dr. McDowell, "I visited her, and, much to my astonishment, found her engaged in making up her bed. I gave her particular caution for the future; and in twenty-five days she returned home in good health, which she continues to enjoy."

It will not be uninteresting here to state that Mrs. Crawford, at the time of the operation performed upon her by Dr. McDowell, lived in Green county, Kentucky, from whence she removed, sometime afterwards, to a settlement upon the Wabash river, in Indiana, where she died, March 30th, 1841, in the 79th year of her age. There was no return of her disease, and she generally enjoyed good health up to the period of her death. She had no issue after the operation. Her youngest child, our worthy citizen, Mr. Thomas H. Crawford, who has kindly communicated to me these facts, was born in 1803, nearly or quite, six years before the operation.

The *second case* was that of a negress. The tumor is stated to have been very large, and so firmly adherent to the bladder and uterus as to render any attempt at extraction perfectly futile. The operator, therefore, contented himself with making a free incision into it with a scalpel, to let out its contents, which were of a thick, ropy and gelatinous character. The incision was of the same length, and made in the same situation, as in the preceding case. Upwards of a quart of blood was lost in the operation. The wound, which was dressed in the ordinary manner, healed without any untoward symptom. The woman remained well for nearly five years, when the tumor began to increase again, and in twelve months it was as large as it was before the operation.

The *third operation* was performed in May, 1816. The subject was a negro woman; and the ovarium, which was much enlarged, could be easily moved from side to side, to the left of which, however, it was adherent. Dr. McDowell made an incision into the linea alba, from an inch below the umbilicus to within an inch of the pubes, and then extended the opening towards the right side, about two inches above the former point, to afford himself more room. He next passed a ligature round the fallopian tube, and 'turned out' the left ovary, which was found to be in a scirrhous condition, and to weigh six pounds. The wound was dressed as in the preceding cases, and the woman was well in two weeks, though the ligature did not come away under five weeks. No mention is made of the manner in which the adhesions were overcome.

Dr. McDowell performed his *fourth operation* in April, 1817, upon a colored woman, from Garrard county, Kentucky, removing a scirrhous ovary, weighing five pounds. The incision was made near the linea alba, but its extent is not mentioned. The ligature slipped from the fallopian tube, after its division, and, in consequence, a great loss of blood took place. Several arteries were then tied; but this not arresting the hemorrhage, a large ligature was passed round the whole stump of the tube, and secured in the most careful manner. Although the woman was much exhausted she happily recovered, but did not fully regain her health. "This, though the smallest ovarium I have ever extracted," says Dr. McDowell, "was much more troublesome to the patient than in any previous case. Besides experiencing severe lancinating pains in the parts, she was seldom able to discharge her urine without getting almost on her head, in consequence of the tumor falling down into the pelvis, and compressing the urethra."

His *fifth* recorded operation was performed by Dr. McDowell on the 11th of May, 1819. The patient, likewise a negress, and the mother of one child, was from Lincoln county, in this State, and was supposed by her physician to be laboring under ascites, as the tumor was very large and fluctuating. After having given her hydrogogue medicines for some time without any benefit, Dr. McDowell tapped her, and drew off thirteen quarts of thick, gelatinous fluid. The operation was repeated in two months, and it was now ascertained, after the matter was all evacuated, that there was a firm substance, of considerable size, which was evidently a dropsical ovary. Some months after this she was again tapped, and the opening was enlarged so as to admit the finger, which was freely used as a probe, that there might no longer be any doubt respecting the true character of the disease. The incision was made on the left side of the median line, down to the tumor, which was found firmly adherent to the parietes of the abdomen and to the intestines, by slender cords, which were easily separated by the hands. The ligamentous bands, attaching the tumor to the uterus, were surrounded by ligatures, after which the tumor was opened, its contents discharged, and the sac extracted. The fluid measured sixteen quarts, and was of a gelatinous character, intermixed with a considerable quantity of hair, and a body resembling very much, in shape, the front tooth of a cow. Violent peritonitis ensued, followed by death on the third day. The uterus and right ovary were perfectly natural, and the ligatures were well applied, and not in a situation likely to injure the adjoining parts.

It will thus be percieved that, of these five cases, three were entirely successful; that one recovered, and remained well for nearly five years, when the tumor recommenced growing; and that one died from peritoneal inflammation within three days after the extirpation of the diseased mass. This success is fully equal to the average success attendant upon ovariotomy in the hands of modern operators. According to the calculations of my friend, Prof. Atlee, of Philadelphia,* founded upon an analysis of upwards of two hundred cases, the rate of mortality for the operation in question is 26½ per cent.

A highly respectable lady, Mrs. O., aged 55 years, of the neighborhood of Nashville, Tennessee, consulted Dr. McDowell, through her professional adviser, Dr. James Overton, in July, 1822, concerning a tumor in the left side of the abdomen, which she had first noticed sometime during the previous December. Being a member of a family inclined to corpulency, she paid no particular attention to it for several months, the more especially as it was free from pain or soreness. The enlargement of the abdomen continued to increase gradually, and early in the following May she felt distinctly, on the left side, and a little below the level of the umbilicus, a small globular tumor, destitute of sensibility, and movable from side to side, as well as from above downwards. About the middle of June, by which time the swelling had considerably augmented in volume, the patient was seized with pains in the back, hips and thighs, much resembling the first pains of parturition. By the use of laxatives, warm bathing and anodynes, these simptoms were subdued, and she enjoyed an interval of ease and health, until the latter part of July, when there was a recurrence of the local

* A Table of all the known operations in Ovariotomy, p. 32. Philadelphia: 1851.

distress, in a more aggravated form, with great tenderness on pressure. The urinary secretion was natural, both as to quantity and quality, and the uterus appeared to be perfectly sound. No fluctuation could be discovered at this time in the swelling; and the integuments of the abdomen were quite lax, except at the site of the enlargement, wnere they were very tense.

When Dr. McDowell visited the patient, in the summer of 1822, the tumor filled nearly the whole of the abdomen, and she had the appearance of a female in the sixth month of utero-gestation. The general health was a good deal impaired, from the absence of sleep, and the presence of fever, and there was a sense of weight and dragging in the pelvis, with acute pain in the swelling, perineum and thighs.

Supposing the disease to consist in a morbid enlargement of the left ovary, Dr. McDowell designed to extirpate it with the scalpel, and for this purpose made an incision from five to six inches in length, along the linea alba, over the most prominent part of the tumor, down to the peritoneum. Having laid bare this membrane, he proceeded cautiously to divide it, intending to make an opening sufficiently large to admit of the removal of the diseased organ. In this, however, he was disappointed; for he had no sooner made his first incision through the peritoneum, than there gushed out, in a full stream, a bloody looking serum, which continued to flow till the sac which had contained it was apparently entirely empty. The quantity thus lost was about one gallon. The edges of the wound were then approximated by several interrupted sutures, light dressings were applied, and the abdomen was encircled by a broad bandage. This constituted the whole of the operative procedure. No attempt was made, or even deemed practicable, to extirpate the diseased organ, inasmuch as it adhered so closely to the peritoneum as to render it impossible to distinguish or separate it from it. Indeed, Dr. McDowell supposed that he was dividing the peritoneum only when the knife penetrated the ovarian sac. The circumstance took him, as well as every one present, by surprise, because it was entirely unanticipated.

The wound continued to discharge matter for some time after the operation, from the lower extremity of the incision, where a tent was kept for that object. The fluid gradually lost its sanious character, and as it diminished in quantity it assumed more and more the appearance of healthy pus. The wound was entirely healed at the end of about five weeks; and the patient, who lived from fifteen to twenty years after the operation, enjoyed excellent health; nor did she, at any subsequent period, suffer any pain or uneasiness which could be justly ascribed to disease of the ovary, or any other organ connected with the uterus.

The interest of this case is heightened by the circumstance that the late President Jackson, who was a near neighbor of the patient, was present at the operation, assisting in holding her hands, and supporting her resolution.

For the above interesting and valuable details, I am indebted to Dr. James Overton, an eminent practitioner of Nashville, Tennessee, who was present at the operation, and who had charge of the case, both before and after the operation by Dr. McDowell, who visited the patient at her own residence.

For the details of the next case I am indebted to my venerable friend, Dr. W. C. Galt, for many years one of the most successful and distinguished physicians of Louisville.

The subject of this case was Miss Plasters, of the neighborhood of this city, who was attacked, in the winter of 1821, with enlargement and pain of the right ovary. The disease gradually increased, and in February, 1823, she was tapped for the removal of the contents of the tumor. The dropsical symptoms, however, soon reäppeared, and believing that excision of the affected organ afforded the only chance of permanent relief, her medical advisers, Drs. Galt and Ragland, requested her to consult Dr. McDowell. I have not been able to obtain any information as to the age of the patient, and the size of the tumor; but from a letter written by Dr. McDowell to Dr. Galt, sometime after the operation, I learn that the enlarged viscus filled the entire abdominal cavity, and that out of nine cases that had presented themselves with this disease, up to the period adverted to, that of Miss Plaster appeared by far the most hopeless. Upon her arrival at Danville, she was so extremely debilitated that it was believed she would hardly be able to sustain the shock of the operation.

The patient having undergone the requisite preliminary treatment, the operation was performed on the 12th of May, 1823. An incision was made into the abdominal cavity, extending the whole length of the linea alba. Finding the tumor so large that it could not be removed entire, a free opening was made into it, discharging about six pints of fluid. The morbid mass was then lifted from its bed, though not without difficulty, a ligature having been previously cast round its foot stalk or uterine attachment. The abdominal cavity having been cleared of blood and water, the edges of the wound were carefully closed, in the usual manner, and the woman put to bed.

The omentum is said to have been much inflamed and thickened, not, as Dr. McDowell supposed, from the effects of the previous tapping, but from organic disease of its own structure. For ten or fifteen days after the operation there was a bloody putrid discharge from the wound, "which," says Dr. McDowell, "I am well assured could arise from nothing but sloughing of the omentum."*

Notwithstanding her debilitated condition before and for sometime after the operation, Miss Plasters entirely recovered. On the 4th of August, less than three months after the removal of the tumor, Dr. McDowell informed Dr. Galt that she was in "perfect health and spirits."

* Letter to Dr. Galt, August 4th, 1823.

